# Dose and gender dependence of chlorine inhalation in a conscious ovine model

**DOI:** 10.1038/s41598-023-48720-2

**Published:** 2023-12-15

**Authors:** Tuvshintugs Baljinnyam, Yosuke Niimi, John R. Salsbury, Satoshi Fukuda, Casey M. Ouellette, Clark R. Andersen, Yasutaka Hirasawa, Donald A. Prough, C. Edwin Garner, Andrew L. Salzman, Perenlei Enkhbaatar

**Affiliations:** 1 Translational Intensive Care Unit, Department of Anesthesiology, The University of Texas Medical Branch, Galveston, TX USA; 2 Department of Pharmacology and Toxicology, The University of Texas Medical Branch, Galveston, TX USA; 3 Department of Biostatistics, The University of Texas MD Anderson Cancer Research Center, Houston, TX USA; 4 Radikal Therapeutics, Beverly, MA USA; 5 Mammoth Preclinical Consulting, Placitas, NM USA; 6 Salzman Group Inc., West Tisbury, MA USA

**Keywords:** Experimental models of disease, Translational research

## Abstract

Characterization of the pathophysiology of ARDS following chlorine gas inhalation in clinically relevant translational large animal models is essential, as the opportunity for clinical trials in this type of trauma is extremely limited. To investigate Cl_2_ concentration and gender-dependent ARDS severity. Sheep (n = 54) were exposed to air or Cl_2_ premixed in air at a concentration of 50, 100, 200, and 300 ppm for 30 min under anesthesia/analgesia and monitored for an additional 48 h in a conscious state. Cardiopulmonary variables and survival endpoints were compared between male and female sheep. Overall there were no significant differences in the responses of female and male sheep except pulmonary oxygenation tended to be better in the male sheep (300 ppm group), and the pulmonary arterial pressure was lower (200 ppm group). The onset of mild ARDS (200 < PaO_2_/FiO_2_ ≤ 300) was observed at 36 h post exposure in the 50 ppm group, whereas the 100 ppm group developed mild and moderate (100 ≤ PaO_2_/FiO_2_ ≤ 200) ARDS by 12 and 36 h after injury, respectively. The 200 ppm and 300 ppm groups developed moderate ARDS within 6 and 3 h after injury, respectively. The 300 ppm group progressed to severe (PaO_2_/FiO_2_ ≤ 100) ARDS at 18 h after injury. Increases in pPeak and pPlateau were noted in all injured animals. Compared to sham, inhalation of 200 ppm and 300 ppm Cl_2_ significantly increased lung extravascular water content. The thoracic cavity fluid accumulation dose-dependently increased with the severity of trauma as compared to sham. At necropsy, the lungs were red, heavy, solidified, and fluid filled; the injury severity grew with increasing Cl_2_ concentration. The severity of ARDS and mortality rate directly correlated to inhaled Cl_2_ concentrations. No significant sex-dependent differences were found in measured endpoint variables.

## Introduction

Chlorine gas (Cl_2_) is a highly reactive toxic gas and widely used in the industrial processes. Its clinical exposure leads to a range of disease manifestations ranging from mild respiratory irritation to acute respiratory distress syndrome (ARDS)^1,2^. According to the American Association of Poison Control Centers, more than 6,300 cases of Cl_2_ exposure were reported in 2019^3^. Upon exposure, Cl_2_ immediately reacts with water on the tissue surface to form hypochlorous acid and oxygen/nitrogen free radical species, damaging cells and cellular components^4,5^. In mild cases, exposure to Cl_2_ may produce mucosal membrane irritation in the upper airway^6,7^; severe cases may lead to local necrosis, epithelial cell layer desquamation^8^, and edema formation in both the upper and lower airways and lung parenchyma^9^. Disruption of airway membranes, alveolar tissue, and endothelial cell death may engender pulmonary edema and ARDS^10,11^.

The general population is at risk of chlorine inhalation exposure from domestic and industrial accidents. A frequent cause of household accidental exposure to chlorine gas is associated with mixing hypochlorite in cleaning products with acid that generates Cl_2_. In industrial usage there is a risk of the accidental release of large amounts of Cl_2_ during transportation, which can cause a mass casualty. Historically, Cl_2_ has been used in the battlefield as an offensive chemical weapon^8^ and there is increasing concern that it will be used again during warfare in the future.

Several studies have reported the effects of Cl_2_ inhalation using various animal models, such as mice, rabbits, pigs, and primates. A few studies have also reported Cl_2_ exposure incidents in armed conflicts or in industrial accidents^8,12–16^. Current emergent treatment options for Cl_2_ exposure remain largely supportive^17^, including the use of humidified oxygen, beta-adrenergic agents for airway obstruction^18^, and corticosteroids^19,20^. To develop a more specific treatment it is important first to benefit from a preclinical model system that recapitulates the clinical symptomatology. Previously Batchinsky et al. reported their results of ovine model Cl_2_ injury^21^ in anesthetized sheep but, a systematic analysis of responses in a sex-dependent manner was not provided. In the present study, we aimed to characterize the severity of cardiopulmonary morbidity and mortality in a Cl_2_ dose- and-sex dependent manner. Additionally, we have investigated cardiopulmonary hemodynamic responses to the various concentrations of chlorine inhalation without anesthetic influence by utilizing a clinically-relevant, conscious ovine model.

## Materials and methods

This study is aimed to determine Cl_2_ concentration and gender-dependent ARDS severity. The study design was an evaluation of results obtained from a clinically relevant, conscious ovine model in the translational intensive care unit at the University of Texas Medical Branch (UTMB).

All animals were treated according to guidelines and study protocols approved by the Institutional Animal Care and Use Committee (IACUC) (protocol #1906049) at UTMB. The study followed the guidelines of the National Institutes of Health (NIH), those of the American Physiological Society for the care and use of laboratory animals, adhered to NIH guidelines, and met the Animal Research Reporting In Vivo Experiments (ARRIVE) criteria.

### Institutional policy guidelines

All procedures were performed in a negative pressure room with a toxic gas evacuation hood in accordance with safety protocols approved by the office of environmental and health safety (EHS).

### Surgical preparation and postsurgical care

A week before the study Merino sheep were surgically instrumented under anesthesia and analgesia, as described previously^22^. Briefly, sheep were anesthetized initially with intramuscular ketamine (16 mg/kg) followed by 10 mg/kg intravenous ketamine administration (KetaVed™; Phoenix Scientific, St. Joseph, MO). Thereafter, anesthesia was maintained with inhaled isoflurane (Piramal Healthcare Ltd. India) provided via mask. Then, the endotracheal tube was inserted and anesthesia was further maintained with inhaled isoflurane (2–5 vol%). A Swan-Ganz thermodilution catheter (model 131F7; Edwards Critical Care Division, Irvine, CA) was inserted into the pulmonary artery through the right jugular vein via 8.5F percutaneous introducer sheath (Edwards Life- sciences, Irvine, CA). The right femoral artery was cannulated and a polyvinylchloride catheter (16-gauge, 24-in., Intracath; Becton Dickinson Vascular Access, Sandy, UT) was placed into the descending aorta. In conjunction with this instrumentation, a silastic catheter was positioned in the left atrium (#508–003, Dow Corning, Midland, MI) via a 5^th^ intercostal thoracotomy. Analgesia was provided by dosing Buprenorphine SR™ (ZooPharm Laramie, WY) for 72 h. Then the sheep were awakened, transferred to the Intensive Care Unit (ICU), and monitored for 5–7 days for surgical recovery.

### Chlorine gas exposure

**Figure 1:**
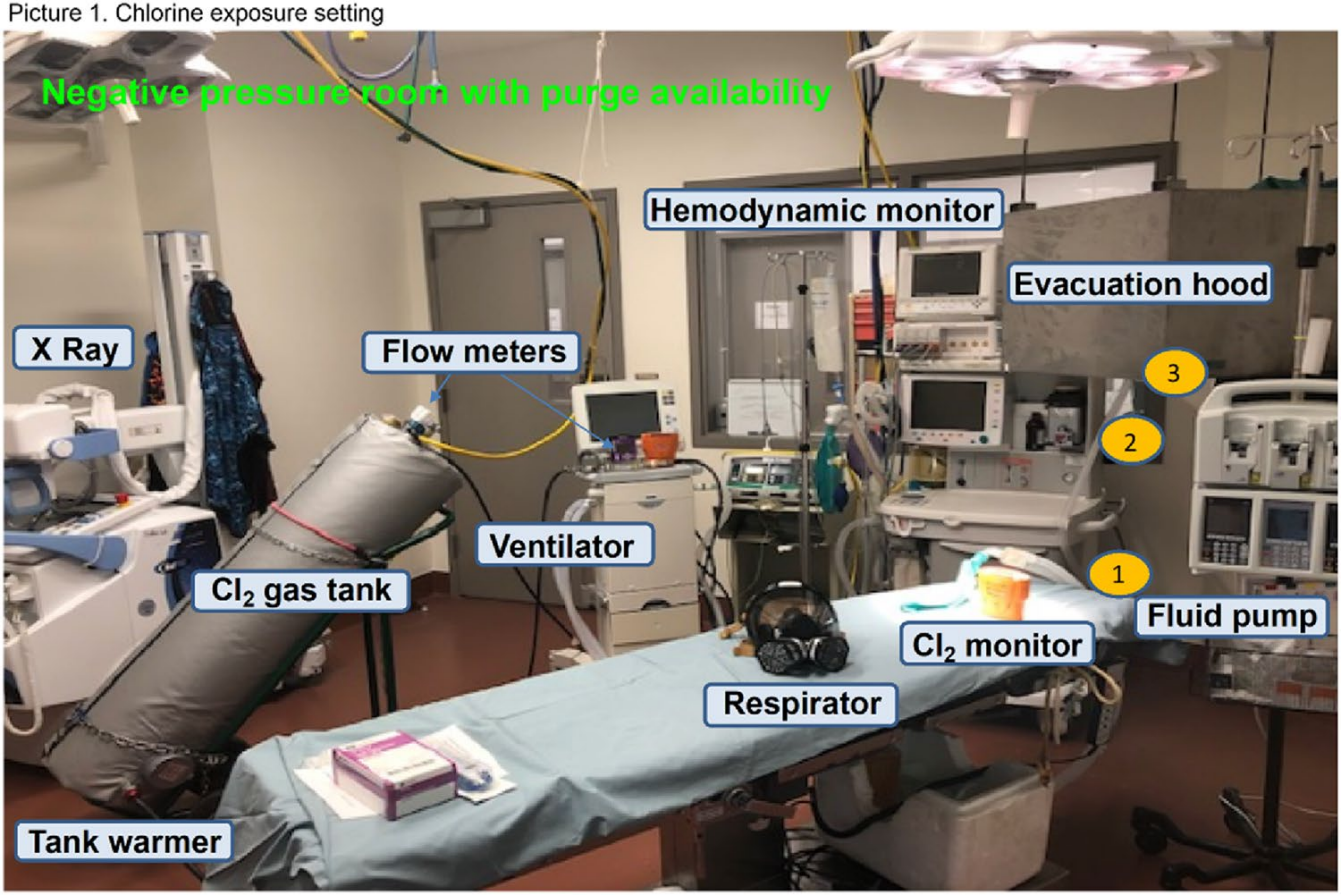
Lab Setting.

Cl_2_ exposure procedures were performed with three investigators in a designated room wearing a fitted, full-facepiece respirator (Scott AV-3000). Two Tetra gas detectors (Crowcon Oxon UK) were used to detect possible Cl_2_ leakage. Additionally, exposure procedures were monitored by a designated scientist through the window (outside of the exposure room) to immediately report to EHS and 911 in case of an emergency. Tanks containing various Cl_2_ concentrations (50, 100, 200, and 300 ppm) premixed with air were purchased from Matheson TriGas, Houston, TX. To assure homogenous gas mixture, gas tanks were preheated up to 40 °C overnight using an electric heating wrap to properly mix the gas in the tank prior to initiating injury. Immediately before the study, sheep were anesthetized with ketamine (10 mg/kg, Bioiche Pharma, Lake Forest, IL) followed by an intravenous continuous infusion of Propofol (Fresenius Kabi USA, LLC, Lake Zurich, IL) and tracheostomy tube placement in the trachea. Analgesia was provided with long-acting buprenorphine (0.01 mg/kg, Zoo Pharm Laramie, WY). Then sheep were mechanically ventilated (Servo 300, Siemens-Elema, Sweden) at a positive end-expiratory pressure (PEEP) of 5 cm H_2_O and a tidal volume (TV) of 15 mL/kg and a respiratory rate of 20 breaths per minute. A Foley catheter was passed through the urethra for continuous quantitation of urinary output. Sheep were exposed to inhalation of air or various concentrations (50, 100, 200, and 300 ppm) of chlorine premixed with air for 30 min (Table 1) via a mechanical ventilator connected to the chlorine or air tank; indicated settings above allow for controlled and comparable delivery of chlorine based on body weight. Prior to beginning, the ventilator and its circuits were checked for detection of possible leaks. The total flow of chlorine/air mixture was determined via a mass flow meter with stainless steel components (Dakota Instrument Company, Orangeburg, NY) placed immediately after the regulator. During gas exposure, arterial PO_2_, PCO_2_, mean arterial and pulmonary arterial pressures, and peak airway pressures were recorded every 10 min. After gas exposure, the Cl_2_ tank was switched to medical air in order to flush out the residue of Cl_2_ in the ventilator system as well as in the sheep airways for 10 min. The diagram of the exposure procedure is shown in Fig. 1.

**Table 1:** Grouping. Cl_2_: chlorine gas; ppm: parts per million; #: number.

Groups	Exposure (30 min)	#Sheep
Female		
Sham	Medical Air	4
Cl_2_ 50 ppm	Cl_2_ 50 ppm	6
Cl_2_ 100 ppm	Cl_2_ 100 ppm	6
Cl_2_ 200 ppm	Cl_2_ 200 ppm	6
Cl_2_ 300 ppm	Cl_2_ 300 ppm	6
Male		
Sham	Medical air	4
Cl_2_ 50 ppm	Cl_2_ 50 ppm	4
Cl_2_ 100 ppm	Cl_2_ 100 ppm	6
Cl_2_ 200 ppm	Cl_2_ 200 ppm	6
Cl_2_ 300 ppm	Cl_2_ 300 ppm	6
Total # sheep		54

After gas exposure animals were transferred to the ICU, where they were placed on mechanical ventilation (Avea, Carefusion, Yorba Linda, CA), resuscitated with lactated Ringer’s solution (2 mL/kg per hour), and allowed to recover from the anesthesia. All animals were monitored continuously for 48 h in a conscious state.

### Grouping

A total of 54 sheep (28 female and 26 male) were included in the study and randomly allocated to five study groups including: (1) Sham (surgically prepared and inhaled medical air) and Cl_2_ exposed: (2) 50 ppm, (3) 100 ppm, (4) 200 ppm, and (5) 300 ppm. The Sham group (both female and male) and the 50 ppm male sheep group had 4 sheep per group, whereas all other groups had 6 sheep (Table 1).

### Measured variables during exposure

During the 30 min exposure, heart rate (HR), mean arterial pressure (MAP), central venous pressure (CVP), pulmonary arterial pressure (PAP), peak airway pressure (pPeak), plateau airway pressure (pPlateau), partial pressure of oxygen in arterial blood, and arterial blood pH were recorded every 5 min.

### Post exposure measured variables

After transferring the animals to the intensive care unit, HR, MAP, CVP, PAP, pPeak, and pPlateau were recorded every 6 h. The PO_2_, PCO_2_ and pH levels were determined in arterial and venous blood at 3 and 6 h after the injury, and thereafter every 6 h for 48 h. PaO_2_/FiO_2_ (P/F) ratio and oxygenation index (OI) were calculated using the standard formulae. Mortality was recorded and presented as survival. Urine was collected from female sheep using an implanted Foley catheter. Chest X-ray images were taken before injury and prior to necropsy using Canon, Rad Pro SM-40 equipped with CXDI Control software NE (Madrid, Spain). Then chest X-rays were examined for scoring the degree of injury by the respiratory physician^23^. The lung injury score was divided into four subgroups based on chest X-ray findings as follows: (0) no consolidation; (1) consolidation confined to one quadrant; (2) consolidation confined to two quadrants; (3) consolidation confined to three quadrants; and (4) consolidation confined to all four quadrants.

### Histology

When moribund criteria were satisfied or at the conclusion of the study, sheep were euthanized and the thoracic and abdominal fluid quantity was measured, lungs were extracted for the determination of W/D ratio as described^24^; and lung, liver, and kidney tissues were fixed in formalin for histological analyses^25,26^. Formalin-fixed lung tissue samples were paraffin embedded, sectioned at 4 µM thickness, and transferred to glass slides. Three slides from each block of tissue were stained with H&E for histopathological assessment. Morphological parameters were scored in blind samples by a board-certified pathologist (Envigo, Israel) using a method of Leustik et al.^25,26^.

### Statistical analysis

All statistical analyses were performed using Graphpad Prism 9. A difference between study groups at each timepoint was analyzed using two-way ANOVA with repeated measures followed by Bonferroni or Tukey’s post hoc tests. The Kaplan–Meier method was used to determine animal survival. Variables are reported as mean ± standard error of mean (SEM). A *p* value of less than 0.05 was considered as statistically significant.

## Results

In the present study, we did not find any statistically significant sex-specific differences in measured variables after exposure to various doses of Cl_2_ except for noted time points where pulmonary oxygenation tended to be better in male sheep (significantly lower oxygenation index at 30 h in 300 ppm group) and PAP was significantly lower at 42 h in the 200 ppm male group. Additionally, MAP was higher in the male 50 ppm group at BL, and 18–42 h. Therefore, we are presenting combined data of both sexes. Gender-separated data can be found in supplemented material.

### Survival

**Figure 2:**
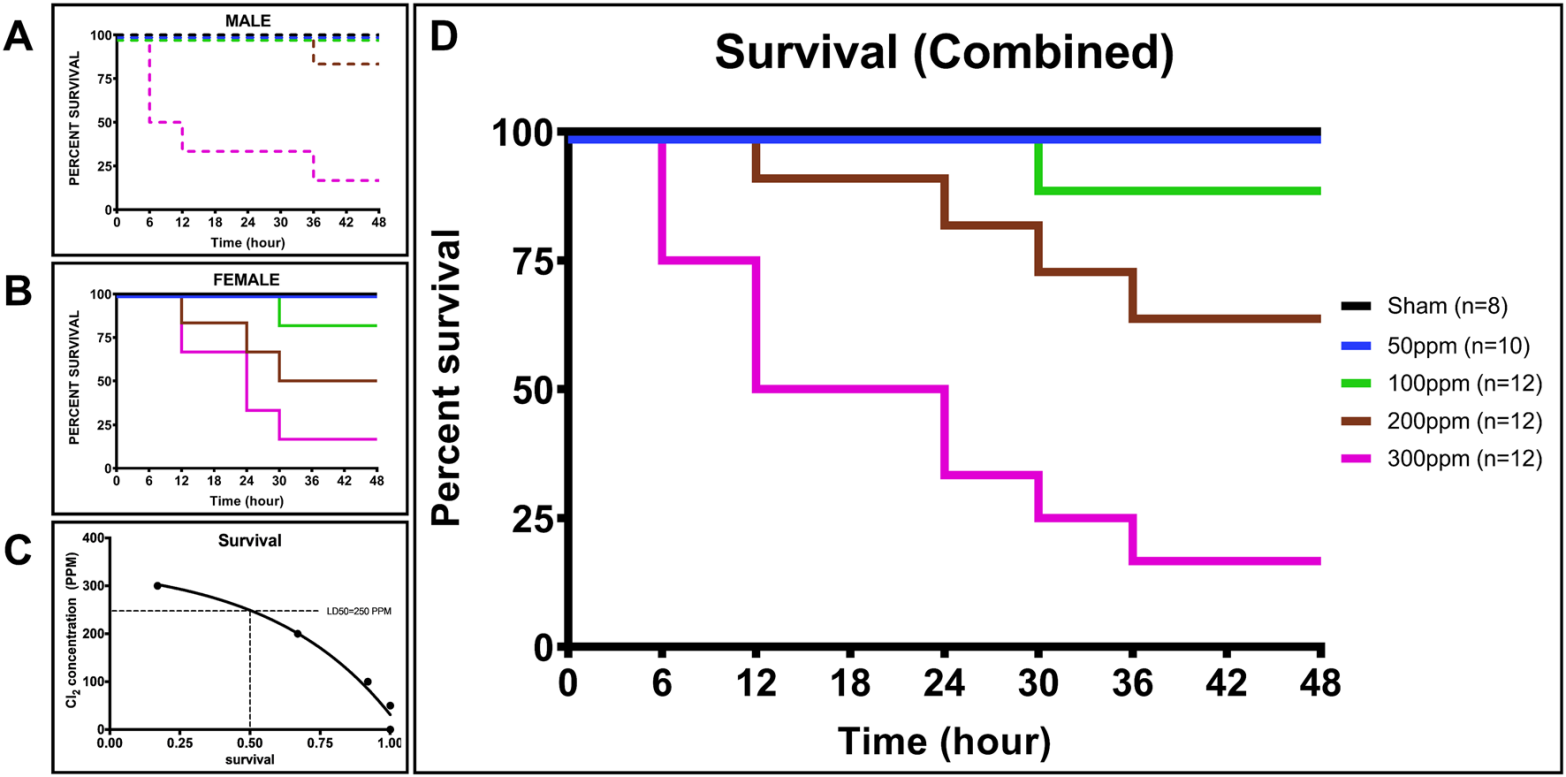
Survival. Percent of survival for (**A**) male, (**B**) female, and calculated (**C**) LD50 concentration (ppm) for male and female combined, and D) combined survival rate for male and female.

Survival decreased in dose-dependent manner in response to Cl_2_ inhalation. Overall survival was similar for both sexes, while there was a trend without statistical significance. A trend shows that, female sheep survival was lower than male, when exposed to lower doses of Cl_2_ (100 and 200 ppm groups), while in the higher dosage (300 ppm group) male sheep survival was lower than female, especially at early time points after exposure (Fig. 2A,B). All animals in the Sham and 50 ppm groups were survived throughout the study. It is noted at 100 ppm Cl_2_ survival percentage was 92.7% at 30 h. The survival percentage in the 200 ppm group was 92.7%, 83.4%, 75.1% and 66.8% at 12, 24, 30, and 36 h, respectively. A drastic decrease in survival was observed in the 300 ppm group at 75%, 50%, 33.4%, 25%, and 16.7% at 6, 12, 24, 30, and 36 h, respectively (Fig. 2D). Following the 30 min Cl_2_ exposure, LD50 was approximately 250 ppm at 48 h (Fig. 2C).

### Cardiopulmonary hemodynamic variables during exposure

**Figure 3:**
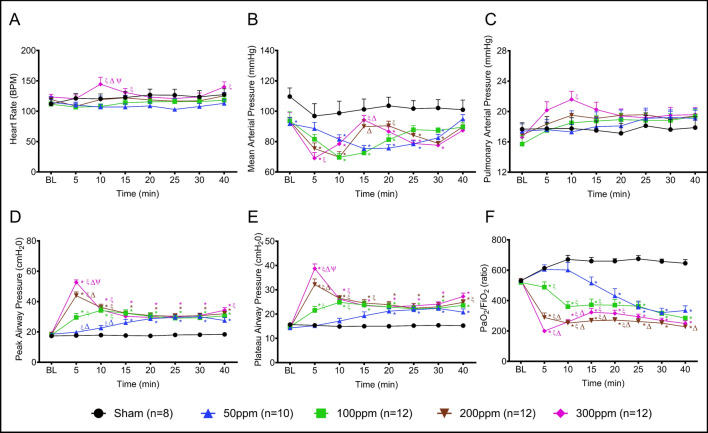
Cardiopulmonary hemodynamics during exposure. (**A**) Heart rate (bpm), (**B**) Mean Arterial Pressure (MAP (mmHg)), (**C**) Pulmonary Arterial Pressure (PAP (mmHg)), (**D**) Peak Airway Pressure (cmH_2_O), E) Plateau Airway Pressure (cmH2O), and F) PaO_2_/FiO_2_ Ratio. *, ξ, Δ, and Ψ indicate significant differences (p < 0.05) compared to Sham, 50 ppm, 100 ppm, and 200 ppm groups, respectively. Data are presented as average value ± SEM.

All animals survived the entire period of 30 min exposure regardless of Cl_2_ concentration. Upon Cl_2_ exposure, the HR remained comparable to baseline (BL) value, except for a significant increase in the 300 ppm group as compared to 50 ppm, 100 ppm, and 200 ppm groups at 10 min after the exposure with a gradual decrease up to 30 min. However, the HR began to increase again after stopping the Cl_2_ exposure, which was significantly higher compared to the 50 ppm group at 40 min (10 min after stopping the exposure) (Fig. 3A).

The mean arterial pressure slightly declined but remained stable in the Sham group during air exposure; however, it sharply decreased in response to Cl_2_ exposure in a dose-dependent manner and recovered to BL level at the end of exposure. In the 300 ppm group, MAP sharply decreased, with its lowest value recorded at 5 min, (p < 0.05 compared to Sham and 50 ppm), and was restored to BL at 15 min. Subsequently, MAP declined again until the end of Cl_2_ exposure (compared to Sham p < 0.05) and was restored to BL level 10 min after stopping gas exposure. In the 200 ppm group, the lowest MAP was recorded at 10 min (compared to Sham p < 0.05) and was restored to BL level thereafter. Afterwards, the MAP gradually declined again until the end of exposure (p < 0.05 to Sham) and was restored to BL level 10 min after stopping exposure. In the 100 ppm group, the lowest MAP was recorded at 10 min (p < 0.05 to Sham), and then gradually restored to BL level by 25 min and remained unchanged until the end of the procedure. In the 50 ppm group, MAP gradually declined, reaching significant difference at 10—30 min (as compared to Sham p < 0.05), and was then restored to BL level at 10 min after stopping exposure (Fig. 3B). At 10 and 15 min after starting exposure, the pulmonary arterial pressure increased in response to the higher dose of Cl_2_, but remained comparable to the BL level (Fig. 3C) in all groups.

Peak airway pressure (PAP; Fig. 3D) sharply increased in the 200 and 300 ppm groups at 5 min following the start of exposure with the highest increase noted in the 300 ppm group (p < 0.05 vs. sham, and all remaining injured groups). Then, the PAP gradually decreased at 10 min, reaching a level below 40 mmHg in both groups and remained unchanged throughout the remainder of the study period. The PAP gradually increased in the 100 and 50 ppm groups, reaching their highest level at 10 min in the 200 ppm group and at 20 min in the 50 ppm group; however, the pressure never exceeded 40 mmHg in these groups. A similar trend was observed in plateau airway pressure upon exposure (Fig. 3E).

Blood oxygen levels decreased in a dose-dependent manner as reflected by the PaO_2_/FiO_2_ ratio at 5 min with a value of lowest to highest in order of 300 ppm > 200 ppm > 100 ppm (compared to Sham p < 0.05). In the 50 ppm group the PaO_2_/FiO_2_ ratio gradually decreased, reaching a significant difference (compared to Sham p < 0.05) at 15 min, and continued to decrease until the end of the procedure (Fig. 3F). In the Sham group the PaO_2_/FiO_2_ ratio remained stable throughout the study period (Some values shown in the Table S1).

### Post exposure cardiopulmonary hemodynamic variables

**Figure 4:**
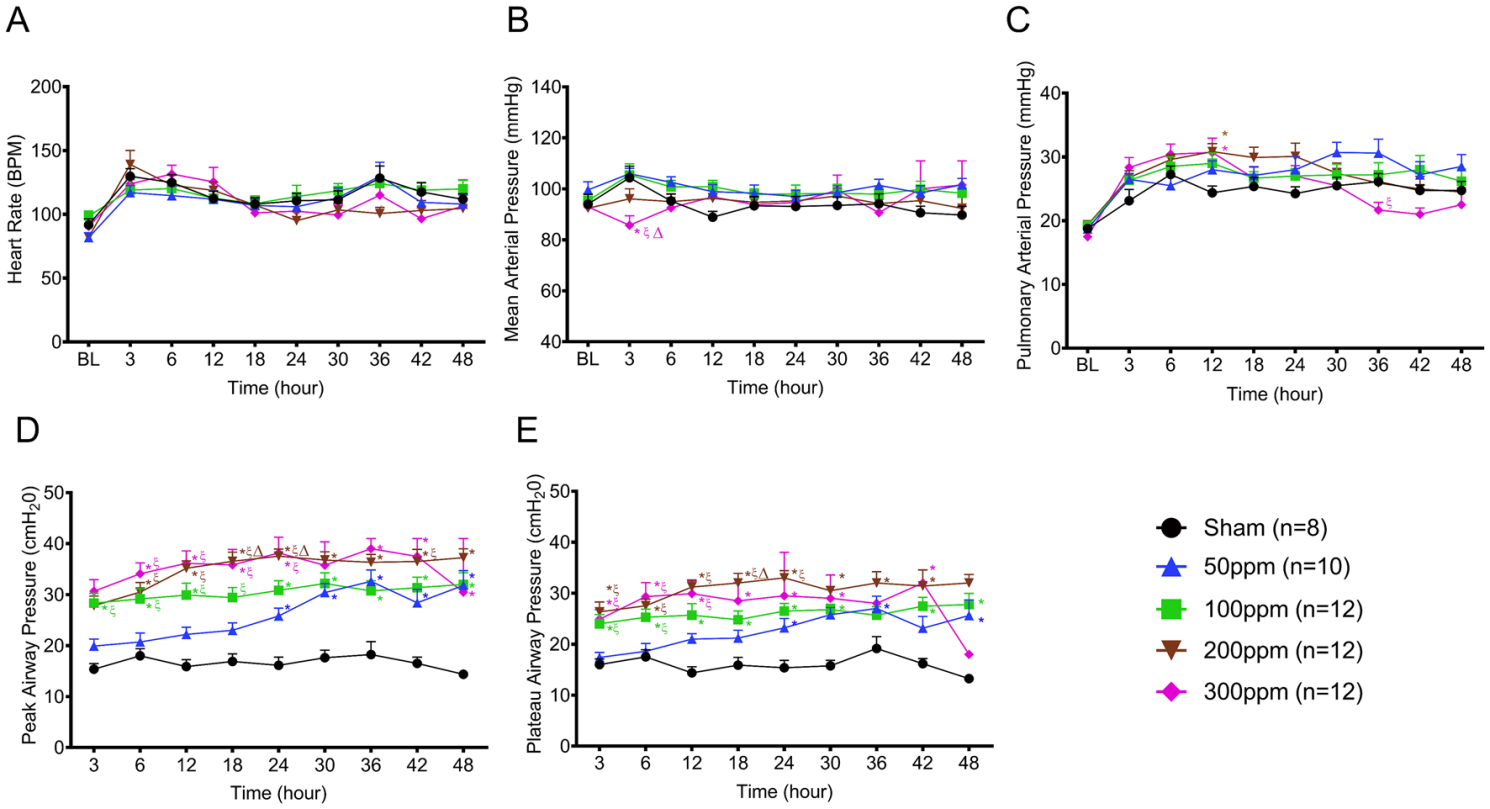
Pulmonary hemodynamics post exposure. (**A**) Heart rate (bpm), (**B**) Mean Arterial Pressure (MAP (mmHg)), (**C**) Pulmonary Arterial Pressure (PAP (mmHg)) (**D**) Peak Airway Pressure (cmH_2_O), and (**E**) Plateau Airway Pressure (cmH_2_O). *, ξ, Δ, and Ψ indicate significant differences (p < 0.05) compared to Sham, 50 ppm, 100 ppm, and 200 ppm groups, respectively. Data are presented as average value ± SEM.

The heart rate slightly increased in all groups over time; however, there were no differences observed between the study groups (Fig. 4A).

The mean arterial pressure slightly increased at 3 h from the BL value (94 ± 3.8 mmHg) approximately by 10 units and remained comparable to BL in the Sham group. A similar trend was observed in 50, 100, and 200 ppm groups where MAP increased by 4–10 units from their respective BL values. Whereas the MAP was significantly lower in the 300 ppm group (85 ± 3.7 mmHg) compared to Sham, 50, and 100 ppm groups at 3 h (p < 0.05) and it remained comparable to the BL level throughout the study (Fig. 4B).

The pulmonary arterial pressure tended to increase starting at 6 h approximately by 8 points from the BL (19 ± 0.6 mmHg) with no statistical significance and was maintained at that level throughout the study in the Sham group. A similar trend was observed in the 50 and 100 ppm groups. A significant increase in PAP was observed in the 200 ppm (31 ± 1.2 mmHg) and 300 ppm (31 ± 2.2 mmHg) groups at 12 h compared to Sham (24 ± 1.0 mmHg) (p < 0.05) and was reduced in the 300 ppm group at 36 and 42 h to (22 ± 1.0 mmHg) and (21 ± 3.5 mmHg), respectively (Fig. 4C).

The peak airway pressure remained unchanged in the Sham group throughout the study period. A low Cl_2_ dose resulted in a gradual increase in pPeak, whereas a higher dose caused a sudden increase. In the 300 ppm group, the pPeak reached a significant increase at 3 h compared to the Sham group and remained higher throughout the study. A similar trend was observed in the 100 ppm and 200 ppm groups, except significant changes were observed at 6 and 3 h, respectively, compared to the Sham group. In the 50 ppm group, a significant increase was noted at 24 h compared to the Sham and remained higher throughout the study (Fig. 4D). A similar trend in pPlateau was observed in Cl_2_-exposed groups, whereas no change was observed in the Sham group throughout the study (Fig. 4E).

### Pulmonary gas exchange (PaO_2_/FiO_2_ratio)

**Figure 5:**
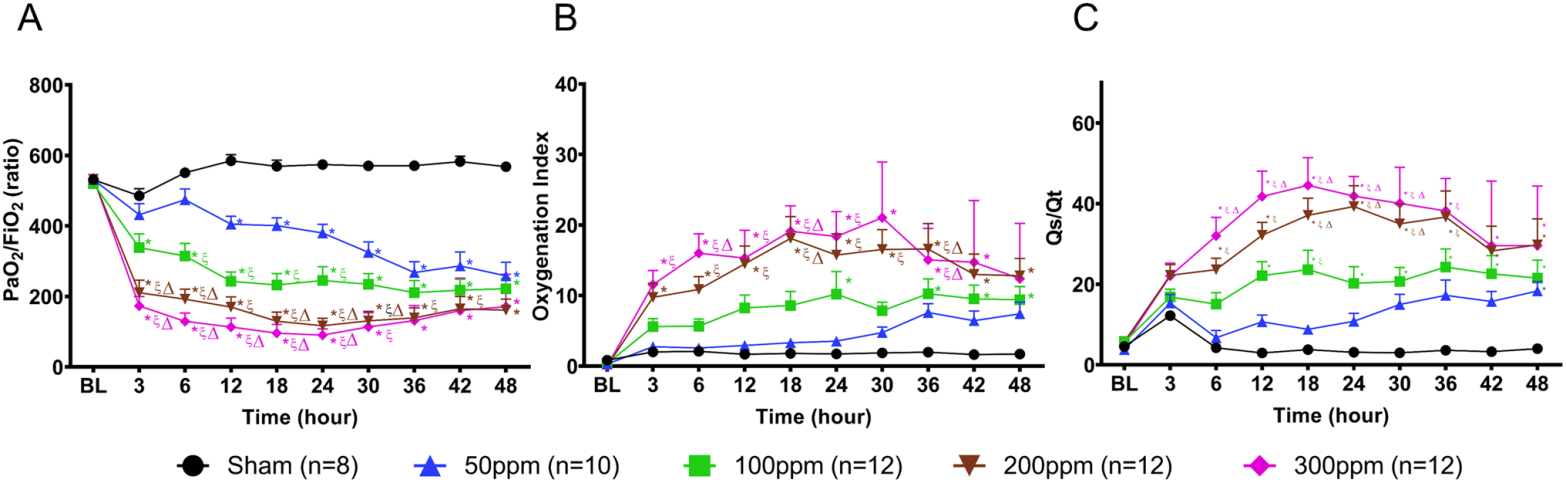
Pulmonary performance. (**A**) PaO_2_/FiO_2_ ratio, (**B**) Oxygenation Index, and **C)** Shunt fraction (Qs/Qt). *, ξ, Δ, and Ψ indicate significant differences (p < 0.05) compared to Sham, 50 ppm, 100 ppm, and 200 ppm groups, respectively. Data are presented as average value ± SEM.

Inhalation exposure to Cl_2_ resulted in a significant dose-dependent reduction in the PaO_2_/FiO_2_ ratio. Pulmonary gas exchange remained comparable to BL levels (PaO_2_/FiO_2_ = 539 ± 9.9) in the Sham group during the study. A significant reduction in oxygenation was observed in the 50 ppm group at 12 h, and in the 100 ppm, 200 ppm, and 300 ppm groups by 3 h compared to Sham. Except for the Sham group, all Cl_2_ exposed animals progressed to ARDS. Mild ARDS developed in the 50 ppm group by 36 h, whereas mild ARDS developed in the 100 ppm group at 12 h and progressed to moderate ARDS by 36 h after injury. In the 200 ppm and 300 ppm groups, moderate ARDS developed by 6 h and 3 h after injury, respectively, and progressed to severe ARDS by 18 h (Fig. 5A).

### Oxygenation index (OI)

In the Sham group, OI remained close to BL throughout the study and increased dose-dependently in Cl_2_-exposed groups. In the 50 ppm group, OI tended to increase at 36, 42, and 48 h. Exposure to 100 ppm resulted in a significant increase in OI at 24 h. At 3 h, OI significantly increased in the 200 ppm group compared to the Sham group, and in the 300 ppm group compared to the Sham and 50 ppm groups (Fig. 5B). The shunt fraction increased in a dose-dependent manner in Cl_2_ exposed groups. In the Sham group, the shunt fraction remained comparable to the level at BL (4.5 ± 0.7). A significant increase was observed in shunt fraction in the 200 ppm group (compared to Sham, and 50 ppm groups) and in the 300 ppm group (compared to Sham, 50 ppm, and 100 ppm groups) groups at 6 h, whereas its increase was noted in the 50 ppm and 100 ppm groups at 48 and 12 h, respectively (Fig. 5C).

### Findings at necropsy

**Figure 6:**
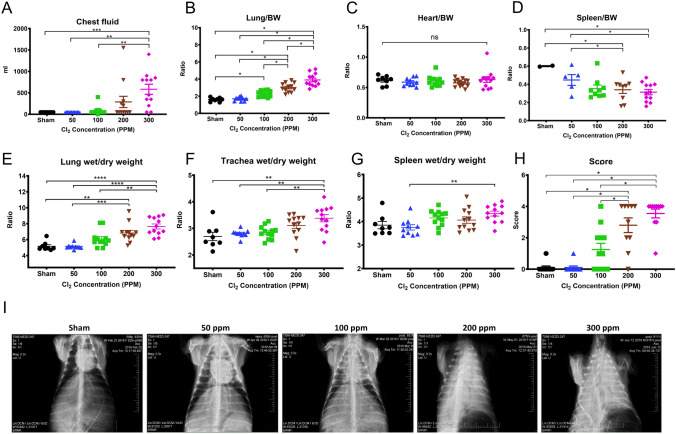
Findings at necropsy. (**A**) Chest fluid, (**B**) Lung/Body weight (BW) ratio, (**C**) Heart/BW ratio, (**D**) Spleen/BW ratio. Wet to dry weight ratio (W/D) for (**E**) lung, (**F**) trachea, and (**G**) spleen. (**H**) Lung damage score, and (**I**) representative chest X-ray images captured immediately before the euthanasia. *, **, ***, **** indicate significant differences with p < 0.05, 0.005, 0.001, 0.0001, respectively. Data are presented as average value ± SEM.

Chest fluid volume tended to increase in a dose-dependent manner. A significant increase was found in the 300 ppm group (550 ± 100 mL) compared to the Sham (50 mL), 50 ppm (50 mL), 100 ppm (75 ± 30 mL) groups, whereas the volume was 275 ± 120 mL in the 200 ppm group (Fig. 6A). Lung wet weight to body weight (BW) ratio was dose-dependently increased (Fig. 6B), whereas heart wet weight to BW ratio remained unchanged regardless to the extent of Cl_2_ exposure (Fig. 6C). Interestingly, spleen wet weight to BW ratio decreased in a dose-dependent manner in injured sheep (Fig. 6D). Lung wet-to-dry (W/D) weight ratio significantly increased in a dose-dependent manner (Sham 5 ± 0.2, 50 ppm 5 ± 0.1, 100 ppm 6 ± 0.2, 200 ppm 6.25 ± 0.3 and 300 ppm 7.5 ± 0.3), where a significant increase found in the 200 and 300 ppm groups compared to the Sham (Fig. 6E). A similar trend of W/D ratio was observed in trachea: Sham (2.8 ± 0.1), 50 ppm (2.9 ± 0.1), 100 ppm (2.95 ± 0.1), 200 ppm (3.15 ± 0.2), and 300 ppm (3.4 ± 0.2) where a significant increase was found in the 300 ppm group compared to the Sham, 50, 100 ppm groups (Fig. 6F). We did not observe a significant change in spleen W/D ratio among study groups, except for a significant increase in the 300 ppm (4.25 ± 0.2) as compared to 50 ppm group (3.75 ± 0.2) (Fig. 6G).

The thoracic X-ray density scoring result correlated with the extent of lung injury as follows: Sham (0.1 ± 0.1), 50 ppm (0.1 ± 0.1), 100 ppm (1.25 ± 0.4), 200 ppm (2.8 ± 0.6), and 300 ppm (3.5 ± 0.3) (Fig. 6H). A significant increase of lung damage score was found in the 200 and 300 ppm groups compared to Sham, 50 ppm and 100 ppm groups (Fig. 6H). Representative chest X-ray images for each group are shown (Fig. 6I).

### Histological findings

**Figure 7:**
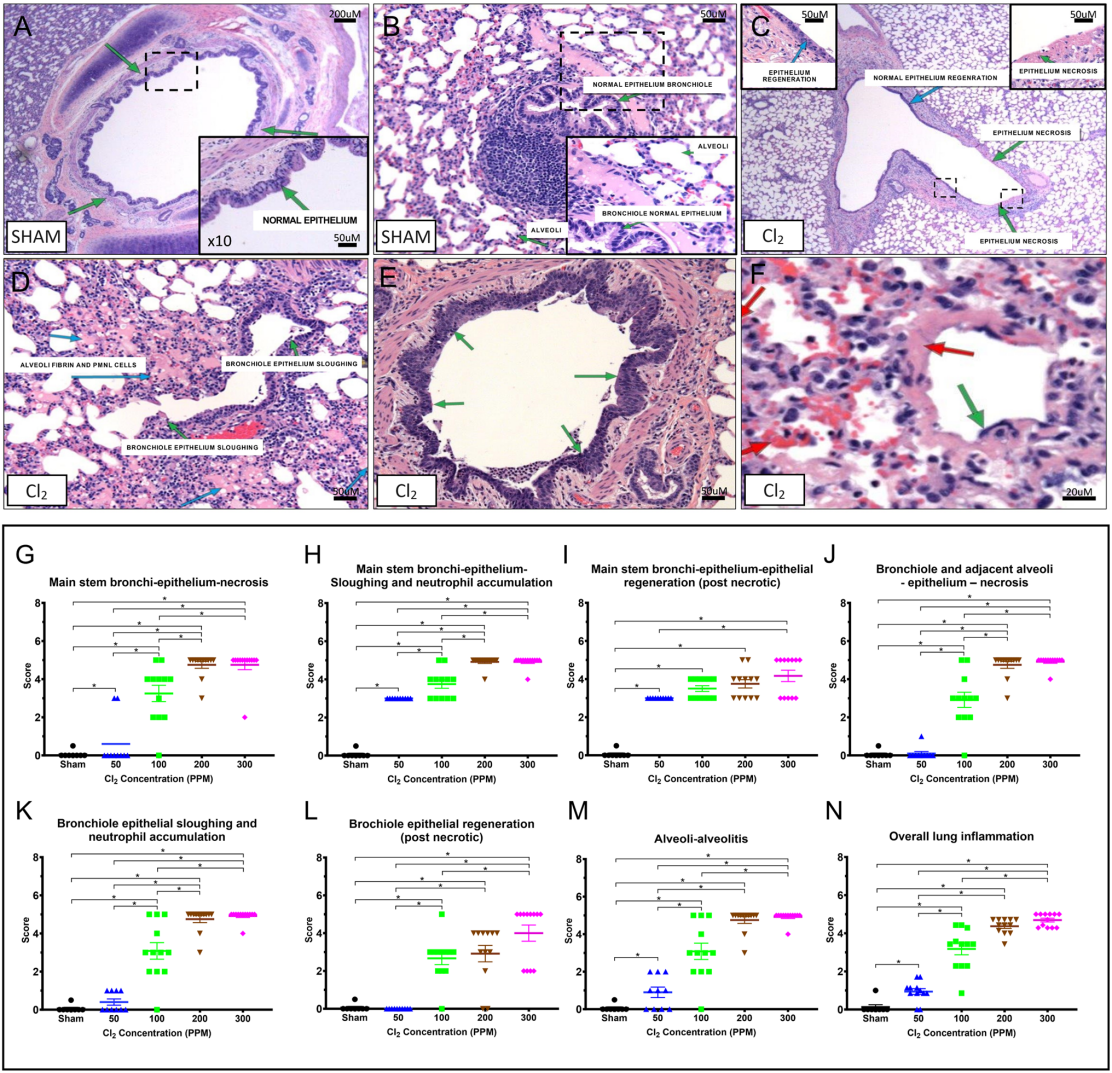
Histological findings and pathological scoring. Normal morphology of (**A**) main stem bronchi, and (**B**) bronchiole epithelium. Tissue damage related to chlorine gas exposure (**C**) main stem bronchi, (**D**) bronchiole epithelium sloughing and alveoli fibrin. Denuded epithelial tissue in (**E**) bronchiole, and (**F**) alveoli. Quantification of main stem bronchi-epithelium (**G**) necrosis, (**H**) sloughing and neutrophil accumulation, (**I**) post necrotic epithelial regeneration. Bronchiole and alveoli epithelium (**J**) necrosis, (**K**) bronchiole epithelial sloughing and neutrophil accumulation, (**L**) post necrotic regeneration of bronchiole epithelium, (**M**) alveoli-alveolitis. Lung inflammation score was calculated and presented in figure (**N**) as overall lung inflammation score. * Indicate significant differences (p < 0.05). Data are presented as average value ± SEM.

Morphological parameters were evaluated in order to reveal the extent of the injury and severity of the lung histopathological changes, using formalin fixed paraffin-embedded, H&E-stained sectional slides. The sham group presented normal morphological evidence with the epithelium lining the mainstem bronchi, and normal epithelium in the bronchioles (Fig. 7A,B). Tissue damage and inflammation were comparable in both sexes. In the 50 ppm group, injury was found mainly in the mainstem bronchi (Fig. 7C), occasionally extending to more distal areas of the lungs (Fig. 7D). The severity of injury was higher in the 100 ppm group compared to the 50 ppm group. In the 200 ppm and 300 ppm groups, the severity of injury and extent of the lesions were greater than 60% of affected tissue. Epithelial tissue was denuded in the bronchiolar and alveolar regions, and neutrophilic infiltrates and high densities of fibrin were present in alveolar spaces in both groups, (Fig. 7E,F). We compared each parameter evaluated between groups and overall scoring generated for each parameter (Fig. 7).

## Discussion

We demonstrate here cardiopulmonary responses to different concentrations of inhaled Cl_2_ in 2 phases: during the 30 min exposure (anesthetized) and during the 48 h post-injury monitoring period (conscious state).

Upon Cl_2_ exposure, the severity of ARDS increased, and survival decreased in a dose-dependent manner, correlating with impairment of pulmonary functions, and intensified tissue damage. The LD50 was estimated to be 250 ppm based on 30 min exposure to Cl_2_ (50–300 ppm) during a 48 h study period.

In addition, for the proper choice of study model to extrapolate clinical conditions, inclusion of both sexes for a drug development study is important due to sex related physiological response. Defensive action of the female hormone and immunosuppressive action by the male hormone could influence experimental outcome. In addition, it is one of NIH policies that requires all biomedical research studies and proposals to consider sex as a biological variable. Also, FDA regulations and guidance acknowledge that understanding the mechanisms of sex differences in medical product development is crucial for regulatory decisions and optimal treatment outcomes. Therefore, we tested whether Cl_2_ exposure results in a sex dependent outcome. As a result, like other studies, we found no significant differences in the survival between the female and male upon Cl_2_ exposure^27^. However, of note, females tended to be more susceptible at lower Cl_2_ concentration (100 and 200 ppm), while males were more resistant. In contrast, at higher Cl_2_ concentration (300 ppm), males tended to die earlier while no death was observed in sham and 50 ppm Cl_2_ groups for both sexes. Additionally, we found that the overall pattern of dynamic changes in cardiopulmonary endpoints was comparable in male vs. female sheep even though some variables such as PAP, and OI were significantly different between the sexes at only a couple of time points, e.g. OI was significantly lower in the male 300 ppm group at 30 h and PAP was significantly lower in the 200 ppm group at 42 h compared to the respective female groups (supplement).

Chlorine gas concentration-dependent pulmonary tissue damage was evidenced by deteriorated pulmonary gas exchange, thoracic X-ray, and histologic analysis findings. Observed tissue damage and symptoms closely resembled what was reported in clinical cases of Cl_2_-exposed patients. For example, data summarized by DOA (1933) for Cl_2_ usage during World War I indicated that subjects were exposed to a high dose of Cl_2_ (estimated > 100 ppm) in the battlefield and experienced a burning sensation in their throat, coughing, feeling of suffocation, and dyspnea accompanied by acute pulmonary edema leading to death within 24 h. Those surviving 48 h mostly recovered, but persisted with bronchitis for weeks and some developed pulmonary emphysema.

Histological findings in sheep indicated that at all tested doses (50–300 ppm) caused necrotic damages observed in the main stem bronchial epithelium. Also, neutrophil accumulation and epithelial sloughing were found in main stem bronchi and bronchiolar epithelium. Alveolar inflammation was triggered at 50 ppm and at 200–300 ppm, its severity was indistinguishable. Interestingly, epithelial necrotic damage was exhibited in bronchiolar and neighboring alveolar epithelium at 100–300 ppm doses, but not at 50 ppm. Our results are in agreement with previous animal studies evaluating Cl_2_ gas inhalational injury^28,29^.

The thoracic-X ray density scoring, chest fluid level, and lung wet-to-dry weight ratio closely correlated with the level of Cl_2_ at higher exposures (200–300 ppm). Additionally, significant damage associated with the pulmonary vessels resulted in edema formation in respiratory organs including the trachea and lung parenchyma. The increase in lung weight to body weight ratio correlated with a higher mortality rate that is most likely secondary to progressive congestion and edema formation with microvascular injury. We did not observe changes in heart weight among groups, indicating that observed changes were limited to respiratory organs including the lung and trachea. However, we cannot rule out the possibility of long-term effects on other organs and respiratory functions^30,31^. Historically, studies of the exposure to Cl_2_ mostly focus on respiratory functions as described previously^32,33^. In the present study, we report a reduced size of the spleen following Cl_2_ inhalation. In adult mammals, including sheep, spleen is the reservoir of most of the red blood cell mass^34^, which can be mobilized from the spleen during hypoxia to increase blood gas storage capacity, resulting in reduced spleen size^35^.

Regarding modeling the clinical disease, non-human primates (NHPs) are considered closest due to genetic background similarity as compared to swine and rodents. However, studies indicate that the ovine model might be closer to human pharmacokinetics and pharmacodynamics as revealed by interspecies comparisons^36^. Also, it is challenging to monitor and collect data from the models using NHPs and pigs, due to their natural behavior, unless they are under deep anesthesia and in an unconscious state. In addition, there are long-standing ethical issues with the use of primates, and recent events have proven that sudden challenges in a global pandemic spread can cause shortages and higher prices for certain model organisms, including NHPs, thereby limiting the studies progress. Taken together we believe that our clinically relevant ovine model is a valuable tool to study human respiratory diseases to further explore and develop treatments, as well as to provide availability of supply. We believe that this study for Cl_2_ inhalation induced lung injury in the ovine model is clinically relevant and provides valuable information that should be considered for any future studies, including but not limited to treatment efficacy and drug development.

### Limitations

This study was focused on characterizing pathophysiological responses and survival in early and later phases of Cl_2_ exposure, therefore the studies at a molecular level to explore mechanistic aspects were not conducted, making this study rather descriptive than mechanistic. The post-injury duration (48 h) was not long enough to reveal the survival at the extended time period. Future studies revealing long-term sequelae of chlorine exposure, including formation of pulmonary fibrotic changes should be considered.

### Clinical implications

This study mimics similar symptoms as those found in Cl_2_ exposed patients. Also, we provide, for the first time to our knowledge, direct evidence characterizing changes in cardiopulmonary hemodynamic and oxygenation changes during Cl_2_ exposure. We also describe the cardiopulmonary pathophysiologic responses during a 48-h post-injury period in a conscious state without anesthetic influence. We believe that our results will help in the future development of effective therapies considering the dose and time-dependent specific pathophysiologic responses in the early acute and chronic post-injury period.

## Conclusions

Our study demonstrated that Cl_2_ inhalation concentration-dependently causes severe ARDS associated with decreased survival. Our study also demonstrated that the severity of injury is comparable between female and male sheep with comparable survival between the sexes.

### Supplementary Information

Supplementary Information.

## Data Availability

Datasets used and/or analyzed during the current study are presented in table or graphic form as main or supplementary figures. Detailed datasets are available from the corresponding author on reasonable request.
