# Supplementary material for: Dose and gender dependence of chlorine inhalation in a conscious ovine model
Source: Sci Rep. 2023 Dec 15;13:22367. doi: 10.1038/s41598-023-48720-2 (PMC10724231; doi:10.1038/s41598-023-48720-2)
Supplement: Supplementary file 1 — Supplementary Information. [file 41598_2023_48720_MOESM1_ESM.pdf]

SUPPLEMENTARY MATERIALS

1. **Table S1.** Cardiopulmonary hemodynamics during exposure………………..………....page 2
2. **Gender separated comparisons (during exposure):**
   2.1. Figure 1S. Mean Arterial Pressure.…………………..……………………...…….page 4
   2.2. Figure 2S. P/F ratio.……………………………………………………….….…..page 5
3. **Gender separated comparisons (post exposure):**
   3.1. Figure 3S. Body Temperature….……………………………………..…………. page 7
   3.2. Figure 4S. Mean Arterial Pressure. ……………………………………...…page 8
   3.3. Figure 5S. Pulmonary Arterial Pressure.……………………………….…...…page 9
   3.4. Figure 6S. Pulmonary Peak Airway Pressure……………………………….....page 10
   3.5. Figure 7S. Pulmonary Plateau Airway Pressure…………………………….....page 11
   3.6. Figure 8S. P/F ratio…………………………………………………….……..page 12
   3.7. Figure 9S. Oxygenation Index…………………………………………………..page 13
   3.8. Figure 10S. Shunt Fraction…….…………………………………………………page 14
   3.9. Figure 11S. Survival…….…………………………………………….……..page 15

Table S1. Cardiopulmonary hemodynamics during exposure

		BASELINE	5MIN	10MIN	15MIN	20MIN	25MIN	30MIN	40MIN	significance	P value
**ANIMAL NUMBER AT TIME POINTS**	SHAM	n=8	n=8	n=8	n=8	n=8	n=8	n=8	n=8	N/A	N/A
	50 ppm	n=10	n=10	n=10	n=10	n=10	n=10	n=10	n=10	N/A	N/A
	100 ppm	n=12	n=12	n=12	n=12	n=12	n=12	n=12	n=12	N/A	N/A
	200 ppm	n=12	n=12	n=12	n=12	n=12	n=12	n=12	n=12	N/A	N/A
	300 ppm	n=12	n=12	n=12	n=12	n=12	n=12	n=12	n=12	N/A	N/A
**HEART RATE**	SHAM	112±9.5	121±7.7	120±8.0	123±9.5	127±9.6	126±9.8	124±10.4	127±9.7	N/A	N/A
	50 ppm	115±4.4	110±4.9	107±4.5	107±5.1	109±6.4	103±3.0	108±4.9	113±5.9	ns	
	100 ppm	112±4.9	107±3.9	108±4.4	114±4.8	115±4.0	116±4.2	116±5.0	118±5.9	ns	
	200 ppm	119±4.9	108±4.9	119±6.0	122±4.1	119±4.6	117±4.9	117±4.9	125±5.6	ns	
	300 ppm	123±4.6	121±6.0	114±11.4$^{\S\Delta\Psi}$	131±6.5§	123±5.2	121±5.5	124±6.5	140±8.2§	ns	>0.05
**MEAN ARTERIAL PRESSURE**	SHAM	110±5.7	97±8.0	99±7.9	101±6.8	104±5.6	102±5.2	102±5.5	101±6.3	N/A	N/A
	50 ppm	115±4.4	110±4.9	107±4.6*	107±5.1*	109±6.4*	103±2.9*	108±4.9*	113±5.9	*	
	100 ppm	112±4.9	107±3.9	108±4.4*	114±4.8*	115±4.0*	116±4.2	116±5.0	118±5.9	*	
	200 ppm	119±4.9	108±4.9*	119±6.0*	122±4.1$^{\Delta}$	119±4.5*	117±4.6*	117±4.9*	125±5.6	*	
	300 ppm	123±4.6	121±6.0*§	144±11.4*	131±6.5$^{\S\Delta}$	123±5.2§	121±5.5*	123±6.5*	140±8.2	*	0.002
**PULMONARY ARTERIAL PRESSURE**	SHAM	17±0.5	17±1.0	17±1.1	17±0.9	17±0.9	18±0.9	17±9.3	17±1.0	N/A	N/A
	50 ppm	24±7.1	25±7.8	24±6.8	23±5.8	23±5.3	24±5.6	25±6.6	28±9.3		
	100 ppm	16±1.1	17±0.8	18±0.6	19±0.8	19±0.8	19±0.9	19±0.9	19±0.9		
	200 ppm	17±0.6	18±0.6	19±0.6	19±0.8	19±0.7	20±0.8	19±1.0	19±1.0		
	300 ppm	17±0.9	20±1.1§	22±1.1	20±1.0	19±0.9	19±1.0	19±1.0	20±0.9	ns	>0.05
**PEAK AIRWAY PRESSURE**	SHAM	18±0.9	18±0.7	18±0.7	18±0.7	18±0.9	18±1.1	18±1.2	18±1.3	N/A	N/A
	50 ppm	18±0.8	20±1.0*	23±1.6*	26±2.2*	29±1.8*	30±1.6*	30±1.8*	27±20*		
	100 ppm	18±1.1	29±2.6*§	34±2.3*§	32±2.3*	30±2.2*	29±1.8*	29±1.6*	31±1.2*		
	200 ppm	18±0.7	44±2.4*$^{\S\Delta}$	36±2.1*§	32±2.1*	31±1.9*	30±1.7*	31±1.7*	32±1.6*		
	300 ppm	19±0.9	53±1.6*$^{\S\Delta\Psi}$	34±1.1*§	30±0.9*	30±1.0*	30±1.1*	31±1.3*	34±1.7*§	ns	>0.05
**PLATEAU AIRWAY PRESSURE**	SHAM	7.2±0.2	7.1±0.1	7.2±0.2	7.2±0.2	7.4±0.2	7.4±0.2	7.5±0.2	7.6±0.3	N/A	N/A
	50 ppm	7.0±0.1	7.4±0.2	7.9±0.3	8.5±0.4	9.0±0.3*	9.1±0.2*	9.3±0.3*	9.0±0.3*		
	100 ppm	8.0±0.7	9.2±0.5*§	10.0±0.4*§	10.0±0.5*	9.4±0.4*	9.1±0.3*	9.3±0.3*	9.7±0.3*		
	200 ppm	7.1±0.1	11.7±0.4*$^{\S\Delta}$	10.7±0.8*§	9.5±0.4*§	9.5±0.4*	9.4±0.3*	9.5±0.3*	9.7±0.4*§		
	300 ppm	7.0±0.3	12.8±0.4*$^{\S\Delta\Psi}$	9.7±0.2*§	9.0±0.2*	9.0±0.2*	9.1±0.2*	9.3±0.3*	10.1±0.4*	ns	>0.05
**PaO_2/FiO_2 RATIO**	SHAM	531±9.9	614±12.3	671±26.8	660±24	660±24	675±21.6	658±15.2	646±24.0	N/A	N/A
	50 ppm	520±11.0	606±30.1	601±49.4	515±40.0*	431±47*	358±39.4*	321±29.0*	335±30.5*		
	100 ppm	520±6.7	488±34.5*§	360±33.4*§	372±38.2*§	370±36.5*	363±26.8*	315±15.5*	285±12.5*		
	200 ppm	532±9.7	288±30.1*$^{\S\Delta}$	252±18.7*$^{\S\Delta}$	267±16.7*$^{\S\Delta}$	271±16.9*$^{\S\Delta}$	262±18.8*$^{\Delta}$	252±16.3*	227±13.3*$^{\Delta}$		
	300 ppm	536±7.3	199±14.3*$^{\S\Delta}$	260±15.0*$^{\S\Delta}$	323±24.4*§	316±21.0*§	294±16.8*	269±15.0*	250±17.4*	ns	>0.05

Table S1. Cardiopulmonary hemodynamics during exposure. Sheep were exposed to indicated dose (SHAM, 50, 100, 200 and 300 ppm) of Cl_2 via endotracheal tube. Then indicated variables were recorded for 30 minutes including every 5 minutes during exposure and additional 10 minutes following end of the exposure. Number of animals per group are indicated as "n". Values ± standard error of mean for heart rate (BPM), mean arterial pressure (mmHg), pulmonary arterial pressure (mmHg), peak airway pressure (cmH2O), plateau airway pressure (cmH2O) and PaO2/FiO2 ratio. The symbols: *, §, Δ and Ψ stand for significant difference compared to baseline, 50 ppm, 100 ppm, 200 ppm, respectively. *$p<0.05$ indicates significant difference between groups, and †$p<0.05$ indicates significant difference compared to baseline.

3. Gender separated comparisons (during exposure)

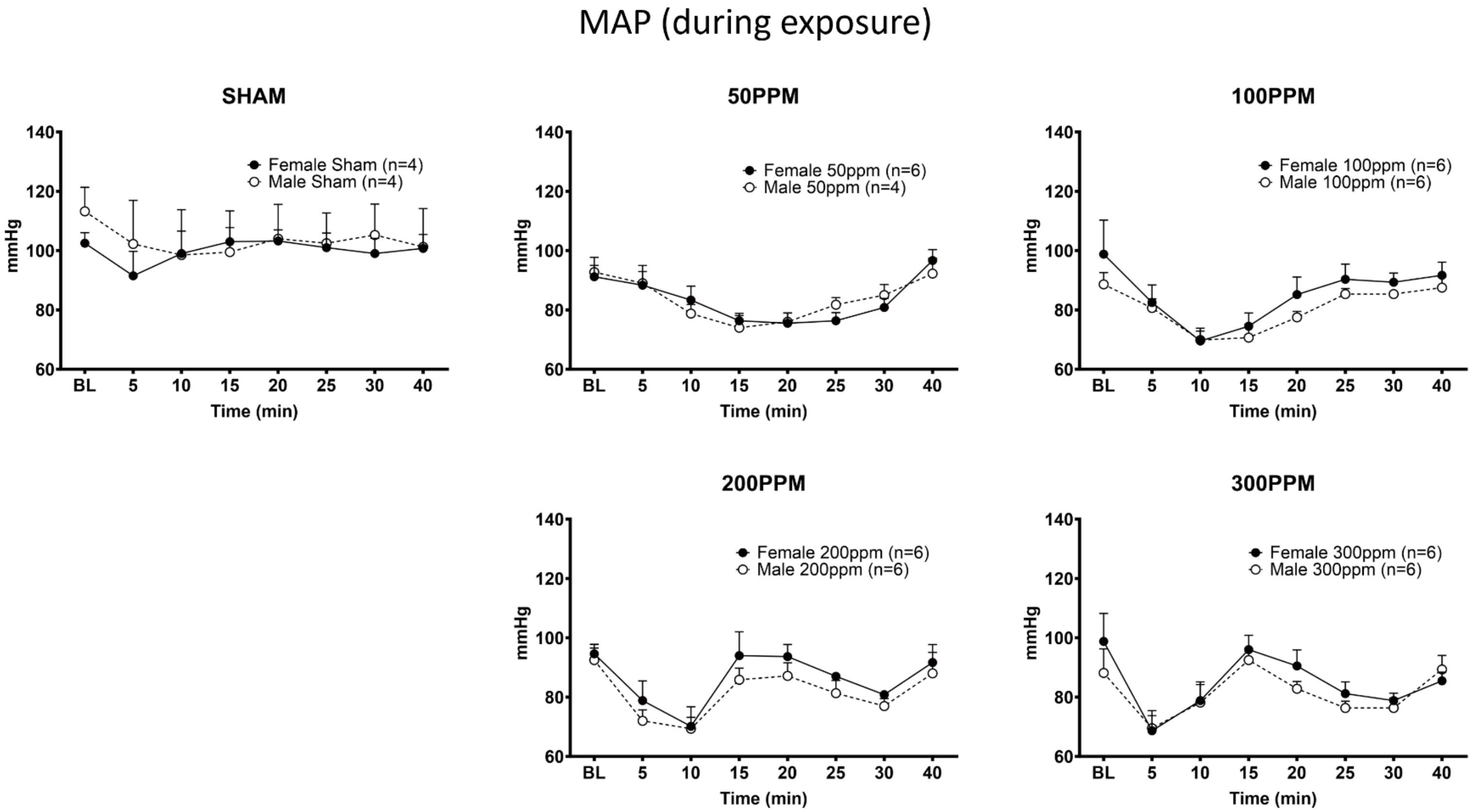

Figure S1. MAP (during exposure). Data are presented as average value ± standard error of mean (SEM). Error bar indicates SEM.

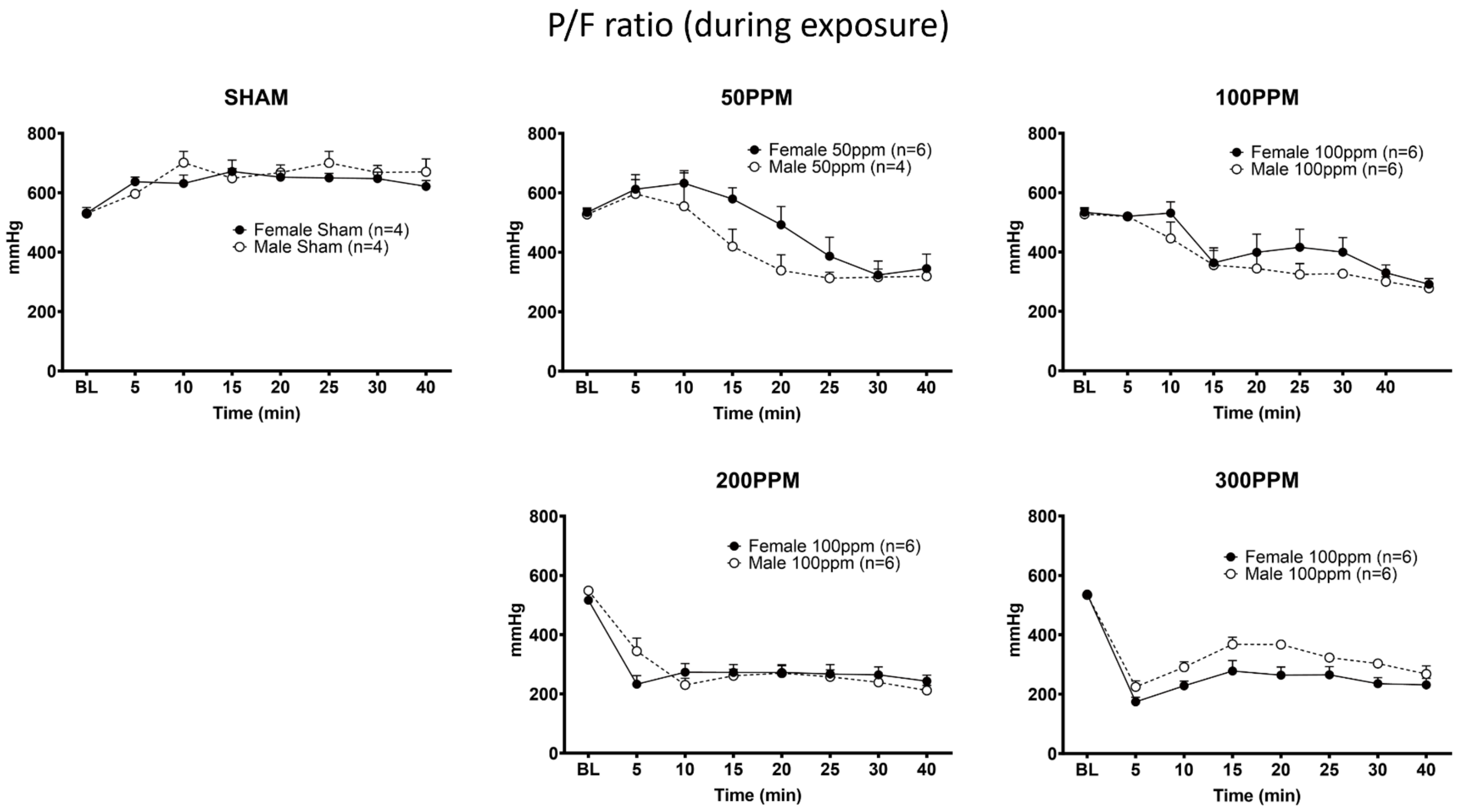

Figure S2. P/F Ratio (during exposure). Data are presented as average value ± standard error of mean (SEM). Error bar indicates SEM.

4. Gender separated comparisons (post exposure)

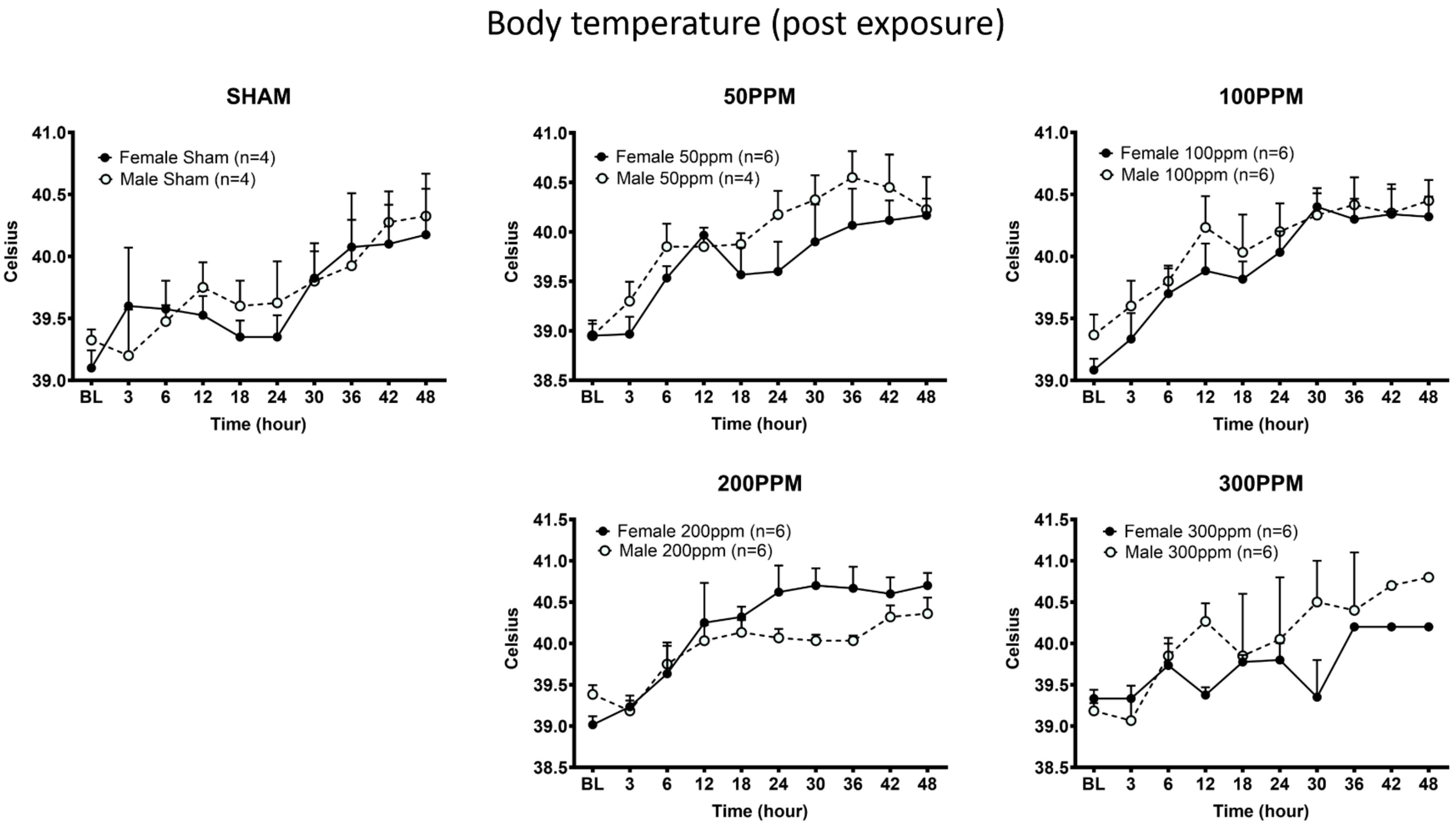

Figure S3. Body Temperature (post exposure). Data are presented as average value ± standard error of mean (SEM). Error bar indicates SEM. No significant difference was found between the sexes.

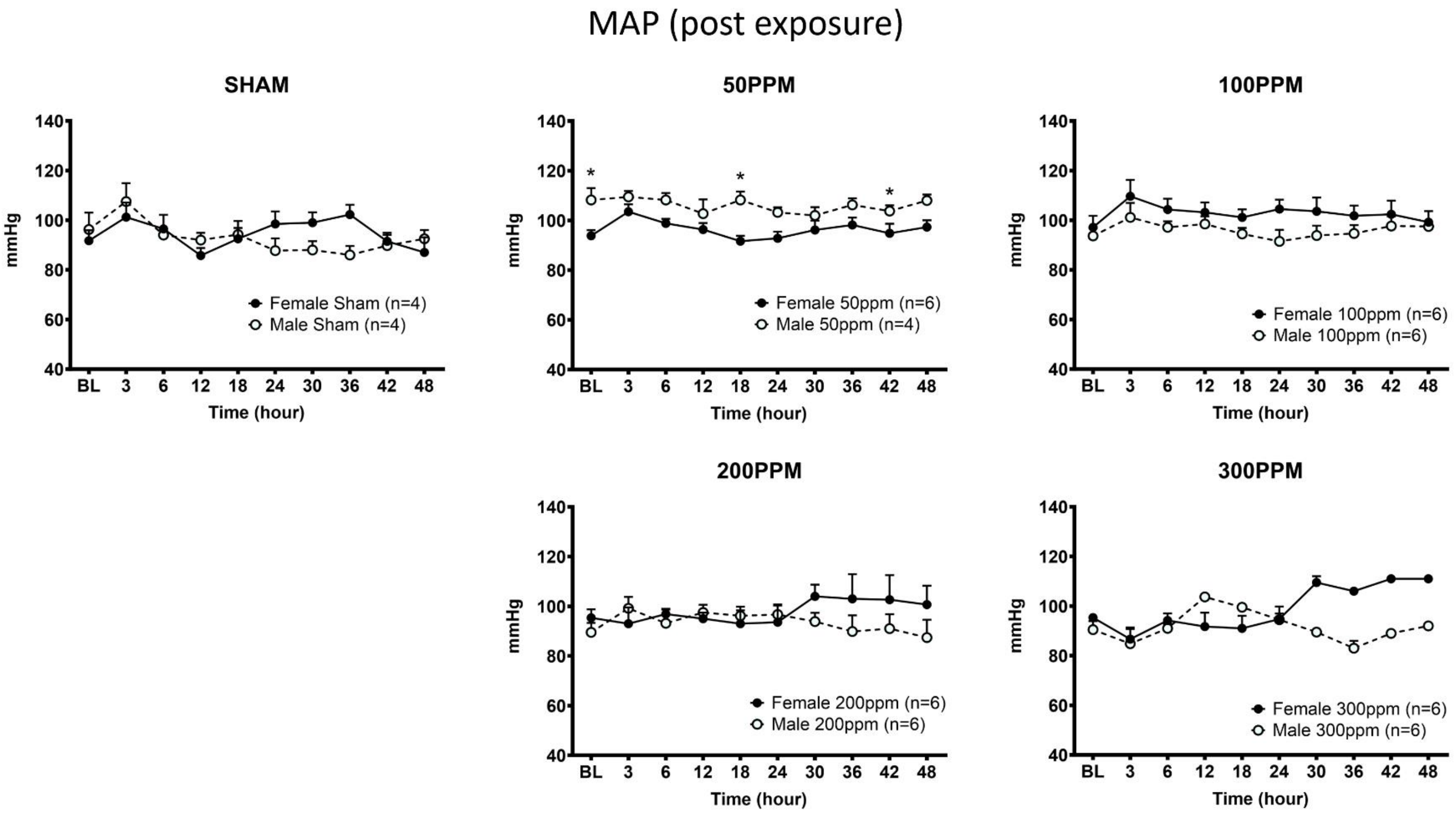

Figure S4. MAP (post exposure). Data are presented as average value ± standard error of mean (SEM). Error bar indicates SEM. *indicates statistically significant (p<0.05) difference between sexes at the indicated timepoint.

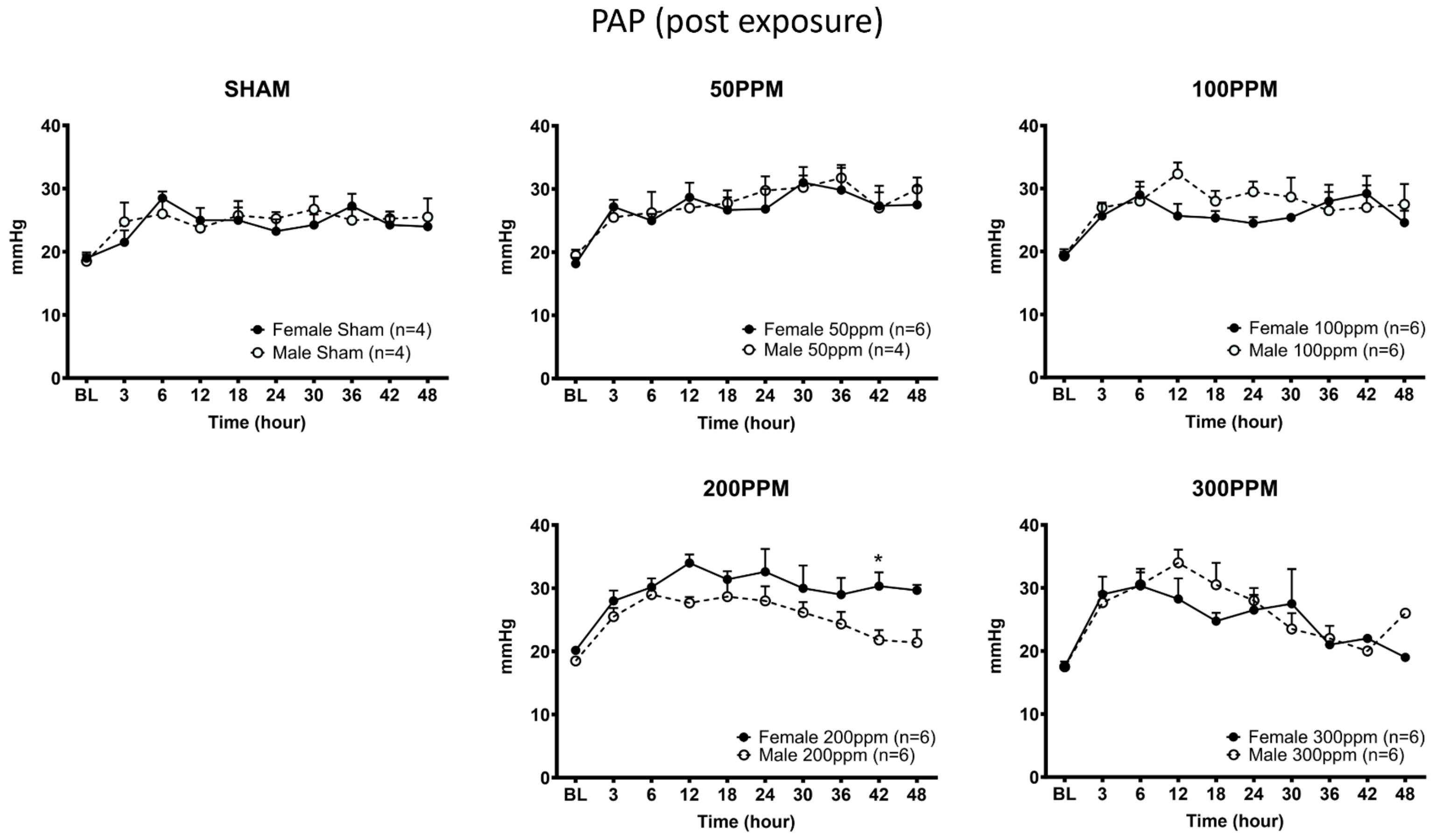

Figure S5. PAP (post exposure). Data are presented as average value ± standard error of mean (SEM). Error bar indicates SEM. *indicates statistically significant (*p*<0.05) difference between sexes at the indicated timepoint.

pPeak (post exposure)

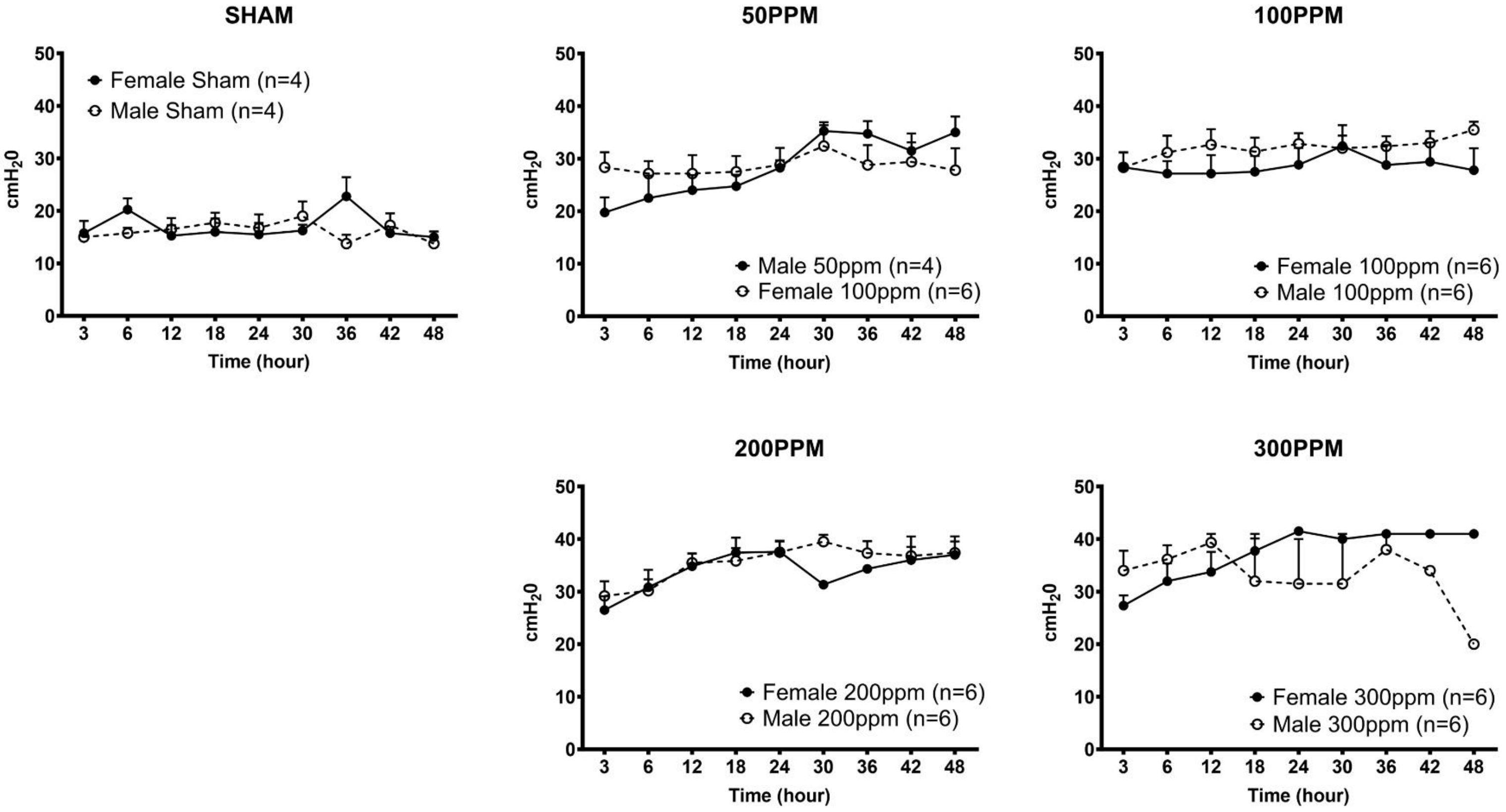

Figure S6. pPeak (post exposure). Data are presented as average value ± standard error of mean (SEM). Error bar indicates SEM. *indicates statistically significant (*p*<0.05) difference between sexes at the indicated timepoint.

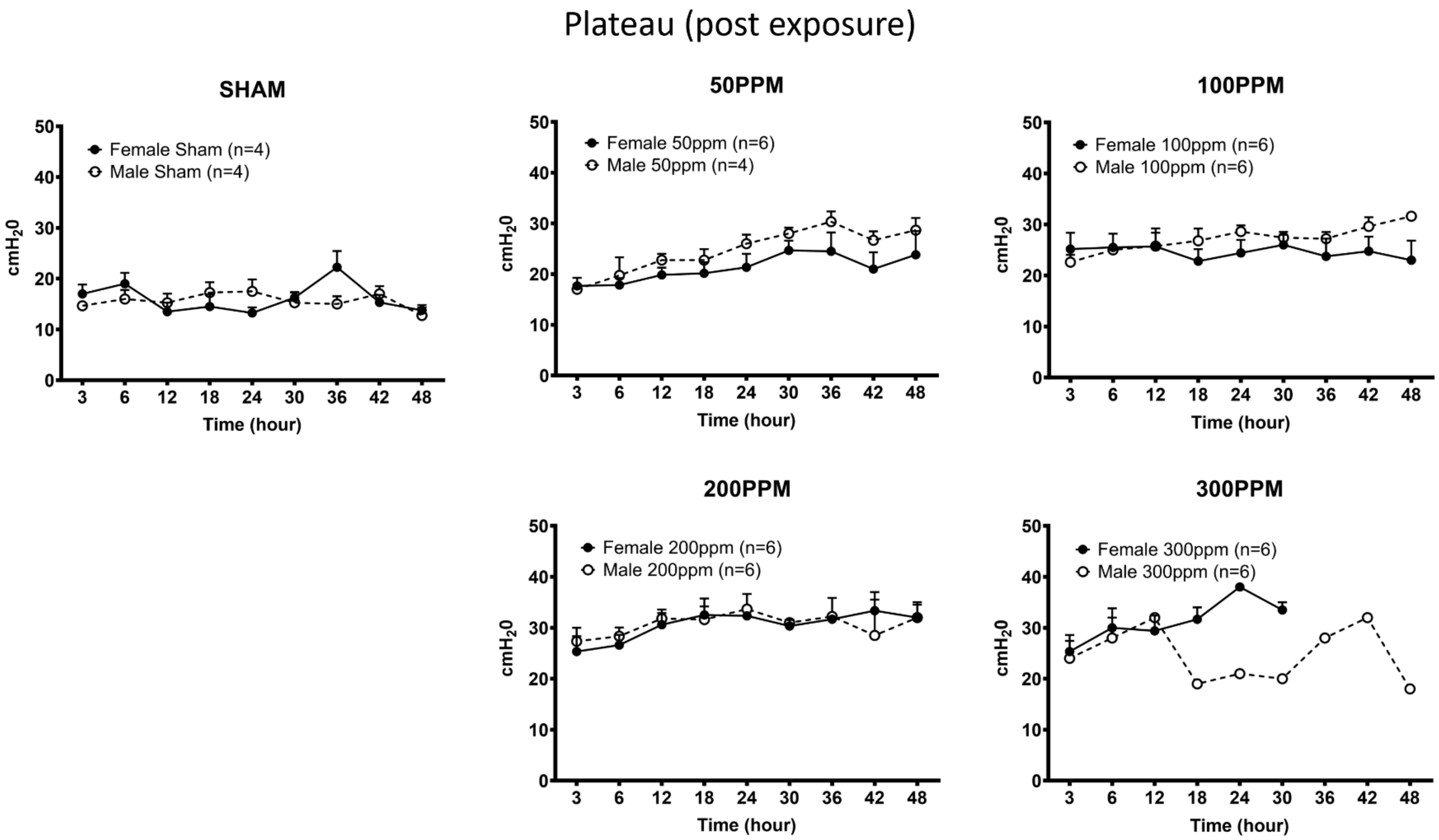

Figure S7. Plateau (post exposure). Data are presented as average value ± standard error of mean (SEM). Error bar indicates SEM. No significant was difference found between the sexes.

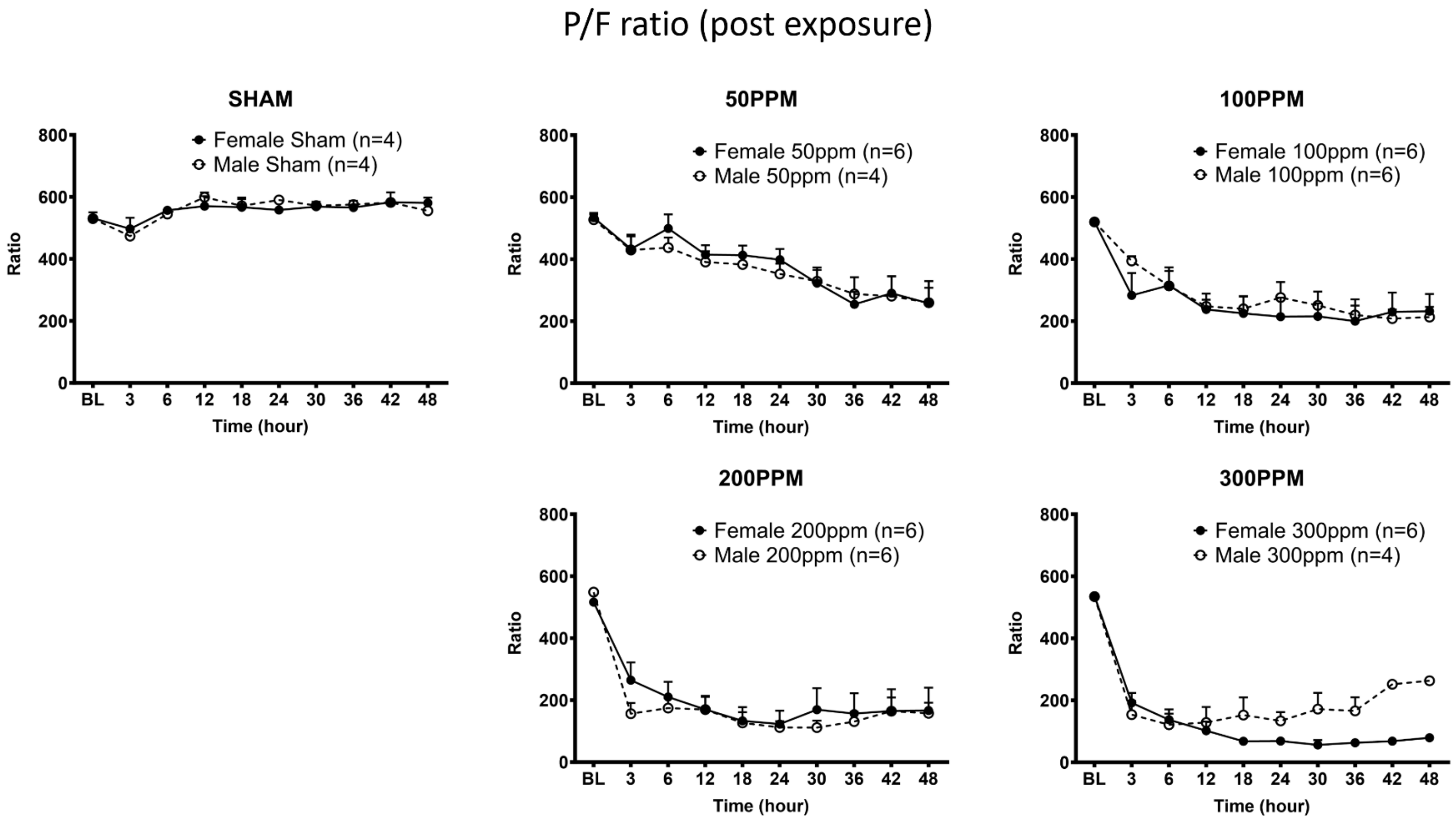

Figure S8. P/F Ratio (post exposure). Data are presented as average value ± standard error of mean (SEM). Error bar indicates SEM. No significant difference was found between the sexes.

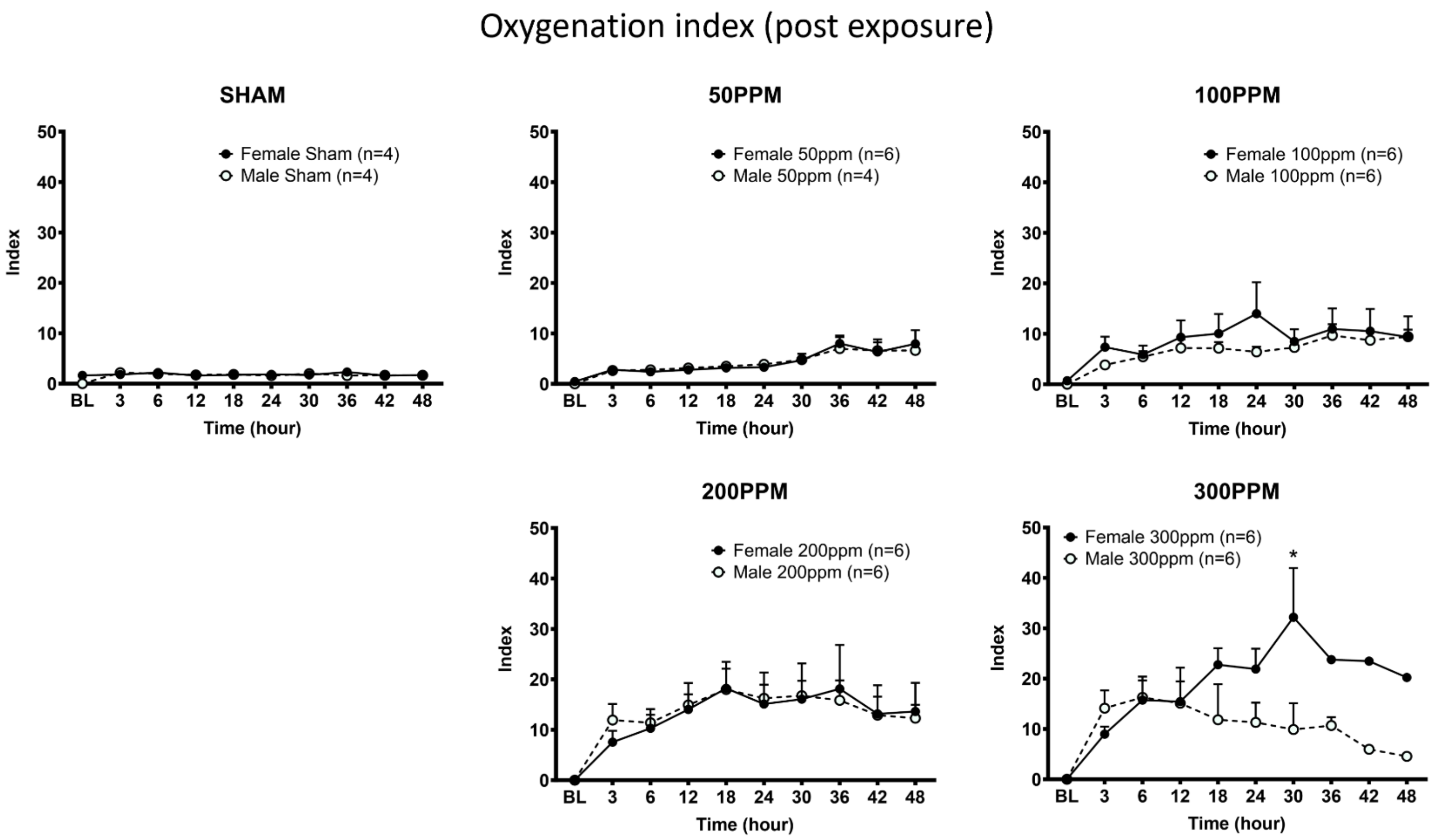

Figure S9. Oxygenation index (post exposure). Data are presented as average value ± standard error of mean (SEM). Error bar indicates SEM. *indicates statistically significant (p<0.05) difference between sexes at the indicated timepoint.

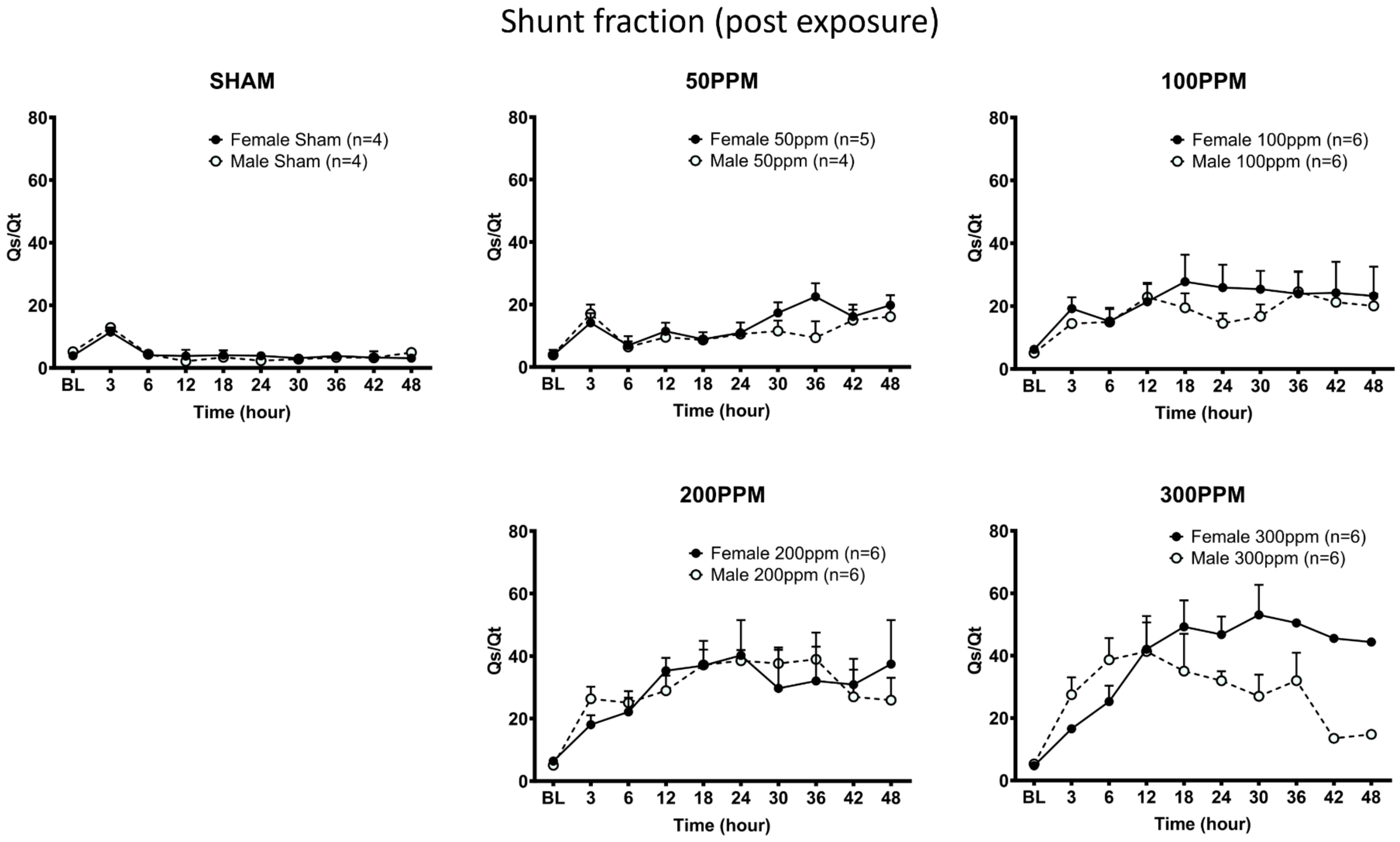

Figure S10. Shunt Fraction (post exposure). Data are presented as average value ± standard error of mean (SEM). Error bar indicates SEM. No significant difference was found between the sexes.

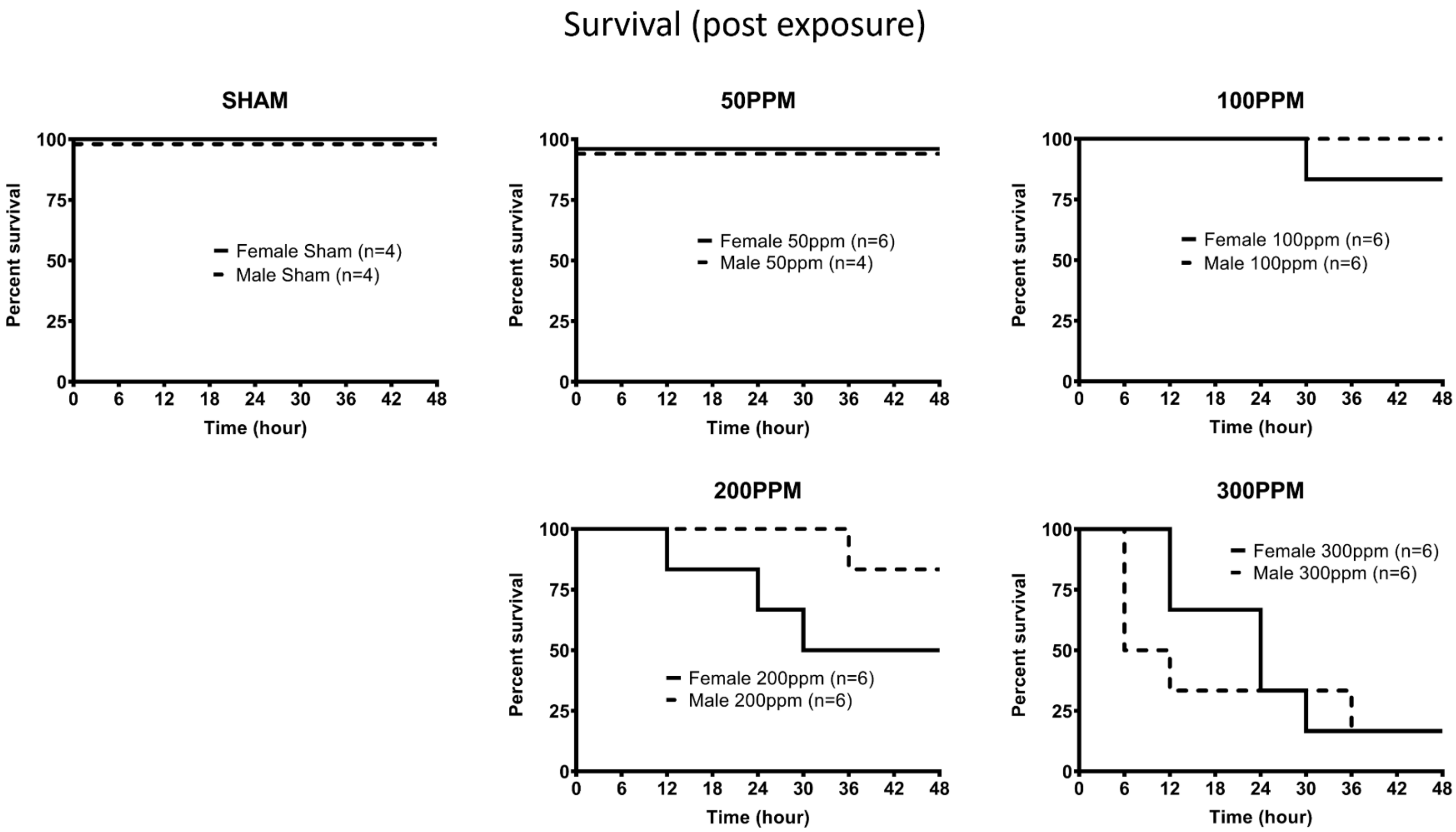

Figure S11. Survival (post exposure). No significant difference found between the sexes (Kaplan-Meier).
